# A Porcine Model of Transvertebral Ultrasound and Microbubble-Mediated Blood-Spinal Cord Barrier Opening

**DOI:** 10.7150/thno.46821

**Published:** 2020-06-19

**Authors:** Stecia-Marie P. Fletcher, Min Choi, Natalia Ogrodnik, Meaghan A. O'Reilly

**Affiliations:** 1 Physical Sciences Platform, Sunnybrook Research Institute, Toronto, ON, Canada.; 2 Department of Medical Biophysics, University of Toronto, Toronto, ON, Canada.

**Keywords:** Focused ultrasound, blood-spinal cord barrier opening, drug delivery, spinal cord therapy, microbubbles

## Abstract

Blood-spinal cord barrier opening, using focused ultrasound and microbubbles, has the potential to improve drug delivery for the treatment of spinal cord pathologies. Delivering and detecting ultrasound through the spine is a challenge for clinical translation. We have previously developed short burst, phase keying exposures, which can be used in a dual-aperture configuration to address clinical scale targeting challenges. Here we demonstrate the use of these pulses for blood-spinal cord barrier opening, *in vivo* in pigs.

**Methods:** The spinal cords of Yorkshire pigs (n=8) were targeted through the vertebral laminae, in the lower thoracic to upper lumbar region using focused ultrasound (486 kHz) and microbubbles. Four animals were treated with a combination of pulsed sinusoidal exposures (1.0-4.0 MPa, non-derated) and pulsed short burst, phase keying exposures (1.0-2.0 MPa, non-derated). Four animals were treated using ramped short burst, phase keying exposures (1.8-2.1 MPa, non-derated). A 250 kHz narrowband receiver was used to detect acoustic emissions from microbubbles. Blood-spinal cord barrier opening was assessed by the extravasation of Evans blue dye. Histological analysis of the spinal cords was used to assess tissue damage and excised vertebral samples were used in benchtop experiments.

**Results:** Ramped short burst, phase keying exposures successfully modified the blood-spinal cord barrier at 16/24 targeted locations, as assessed by the extravasation of Evans blue dye. At 4 of these locations, opening was confirmed with minimal adverse effects observed through histology. Transmission measurements through excised vertebrae indicated a mean transmission of (47.0 ± 7.0 %) to the target.

**Conclusions:** This study presents the first evidence of focused ultrasound-induced blood-spinal cord barrier opening in a large animal model, through the intact spine. This represents an important step towards clinical translation.

## Introduction

Diseases and disorders of the central nervous system (CNS) pose a unique challenge to conventional methods of drug delivery due to the presence of the blood-brain and blood-spinal cord barriers (BBB and BSCB) 1,2. These are neurovascular units, characterized by a physical barrier, consisting of non-fenestrated endothelial cells with tight junctions, and a transport barrier due to membrane transporter proteins and vesicular mechanisms 1. Together, these limit both transcellular and paracellular transport of foreign molecules, including therapeutics, from blood into the CNS parenchyma. Consequently, only a small class of drugs with low molecular weight (< 500 Da) and high lipophilicity are able to cross these barriers in therapeutically relevant quantities 3.

One promising method for bypassing these barriers is the use of focused ultrasound (FUS) in conjunction with intravenously injected ultrasound contrast agents or microbubbles (MBs) 4. When MBs in the vasculature are sonicated with FUS, they oscillate and interact with vessel walls leading to a range of bioeffects 5 which result in the localized and transient opening of the CNS barriers. Because MBs are strong nonlinear scatterers of ultrasound, acoustic emissions from oscillating MBs in the vasculature can be monitored to assess treatment efficacy and potential for tissue damage 6,7. For pulsed FUS sonications, on the order of milliseconds, the presence of spectral frequency signatures have been well characterized: those associated with stable MB oscillation, like harmonic emissions, have been linked to successful barrier opening, while those associated with inertial cavitation and MB collapse, like broadband emissions, have been linked to widespread tissue damage. The understanding of the correlation between spectral signatures and tissue bioeffects has allowed the development of real-time acoustic emissions based control algorithms to promote safe and effective treatments 8-12.

In the brain, there have been a large number of preclinical studies demonstrating the increased permeability of the BBB to tracers and therapeutic agents following FUS + MB treatments 13-19. Recently, FUS-induced BBB opening (BBBO) has reached the stage of clinical trials for treatment of primary brain tumours 20 and early to moderate stage Alzheimer's disease 21. There has been markedly less effort towards translating this method to the spinal cord. However, in the past decade, preliminary research in animal models has indicated that the BSCB can be modified using FUS + MBs, similarly to the BBB 22-27. To date, these studies have been limited to transvertebral studies in rodents 22-26, in which the thickness and density of vertebral bone is not comparable with humans, or in rabbits, which have included pre-treatment laminectomies 27. Although a laminectomy may be appropriate in cases of injury or focal tumors, there is a need to develop non-invasive approaches through the intact bone for this treatment to be relevant to diffuse disease, such as leptomeningeal metastases 28 or neurodegenerative diseases, like multiple sclerosis 29 or amyotrophic lateral sclerosis 30, where treatment along large portions of the neuroaxis is required.

The challenges that must be overcome to safely and efficiently deliver FUS and detect acoustic emissions through the human spine are non-trivial. Developing robust solutions to combat these challenges is essential for clinical translation. Phased arrays tailored to the spinal geometry may provide an opportunity to overcome the low FUS transmission (~ 31 ± 17% at 514 kHz) and high levels of focal distortion that arise as sound propagates through vertebral bone 31,32. At the submegahertz frequencies needed to minimize attenuation and aberration 33, the focal size is large compared to the size of the spinal canal. Due to reflections off the interior walls of the spinal canal, this situation is prone to the formation of standing waves which can compromise treatment safety 34. To address this, a dual aperture, cross-beam approach through the vertebral laminae using short burst, phase keying (SBPK) exposures has been proposed 35. SBPK FUS exposures can be modified to leverage pulse inversion (PI) 36 to enhance detection of MB emissions through the intact spine 26. SBPK FUS exposures have been shown to successfully open the BSCB in small animals, by our group 26.

An important step towards clinical translation is testing these proposed methods in a clinically relevant, large animal model, where spinal geometry is comparable with humans. The porcine spine has previously been identified as a good anatomical model of the human spine in the mid to low thoracic region 37.

In this work, we present a model of FUS-induced BSCB opening (BSCBO) through the intact porcine spine, using SBPK exposures.

## Methods

### Ultrasound generation

Two in-house fabricated, spherically focused, piezocomposite lead zirconium titanate (PZT) transducers with a center frequency (f_0_) of 486kHz were used for these experiments (DL-47 composite elements sourced from DeL Piezo Specialties, LLC, FL, USA; aperture diameter = 5 cm; nominal focal length = 10 cm). Each transducer had a 5 mm bore into which acoustic receivers could be inserted. The transducers were driven using a dual-channel arbitrary function generator (AFG 31000 Series, Tektronix, Beaverton, OR, USA) and two 53 dB RF power amplifiers (NP-2519, NP Technologies Inc., Newbury Park, CA, USA). An in-house fabricated unfocussed, narrowband 5 mm PZT receiver (DL-47 element sourced from DeL Piezo Specialties) with a center frequency of 250 kHz (near the subharmonic (f_0_/2)) was used in this experiment.

**Figure 1:**
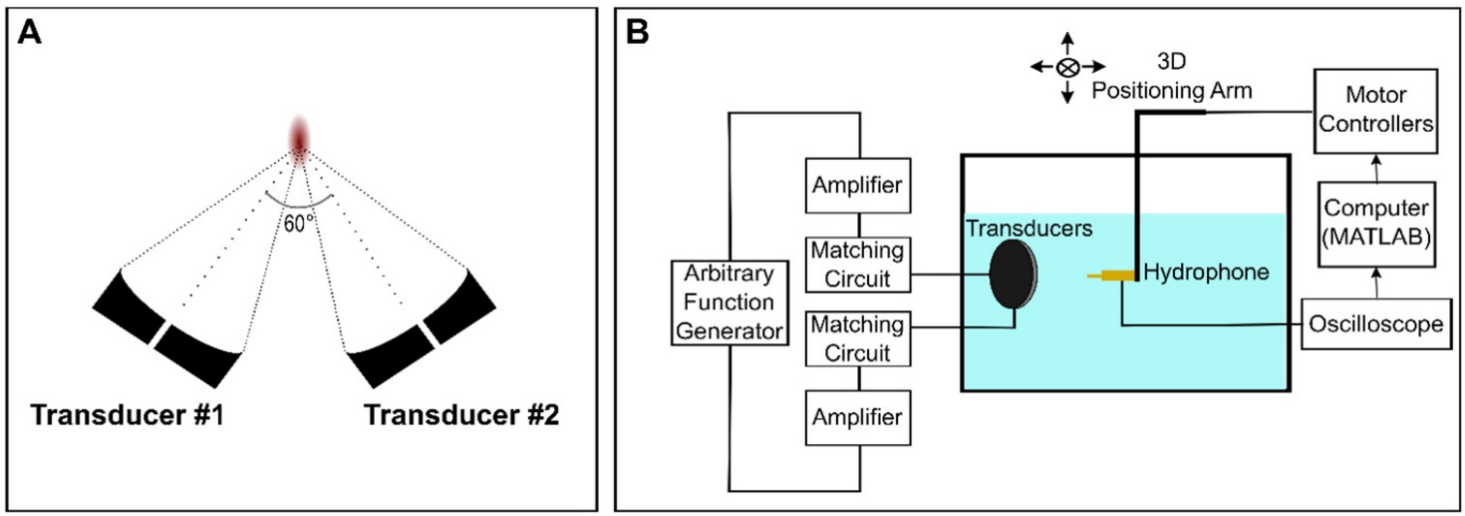
Schematic diagrams of (**A**) the confocal, dual-aperture configuration and (**B**) the set up used for transducer calibration in a scan tank.

In the confocal, dual-aperture configuration (Figure 1A), using SBPK pulse trains, the focal dimensions (70% pressure profile) were 8.4 × 4.5 × 4.7 mm^3^. The pressure at the focus of the transducers for a given driving voltage was calibrated by performing field scans in a tank of degassed and deionized water using a 0.5 mm polyvinylidene difluoride (PVDF) hydrophone of known calibration (Precision Acoustics, Dorchester, UK) mounted on a 3D positioning stage (Velmex Inc., Bloomfield, NY, USA). The 3D positioning stage was controlled using MATLAB. Hydrophone data were displayed using a mixed-domain oscilloscope (MDO 3014, Tektronix, Beaverton, OR, USA) and subsequently uploaded to a PC and stored for processing in MATLAB. A schematic diagram of the set up used for transducer calibration is shown in Figure 1B. Transducers were calibrated using both long (30 cycles), sinusoidal exposures and SBPK exposures.

**Figure 2:**
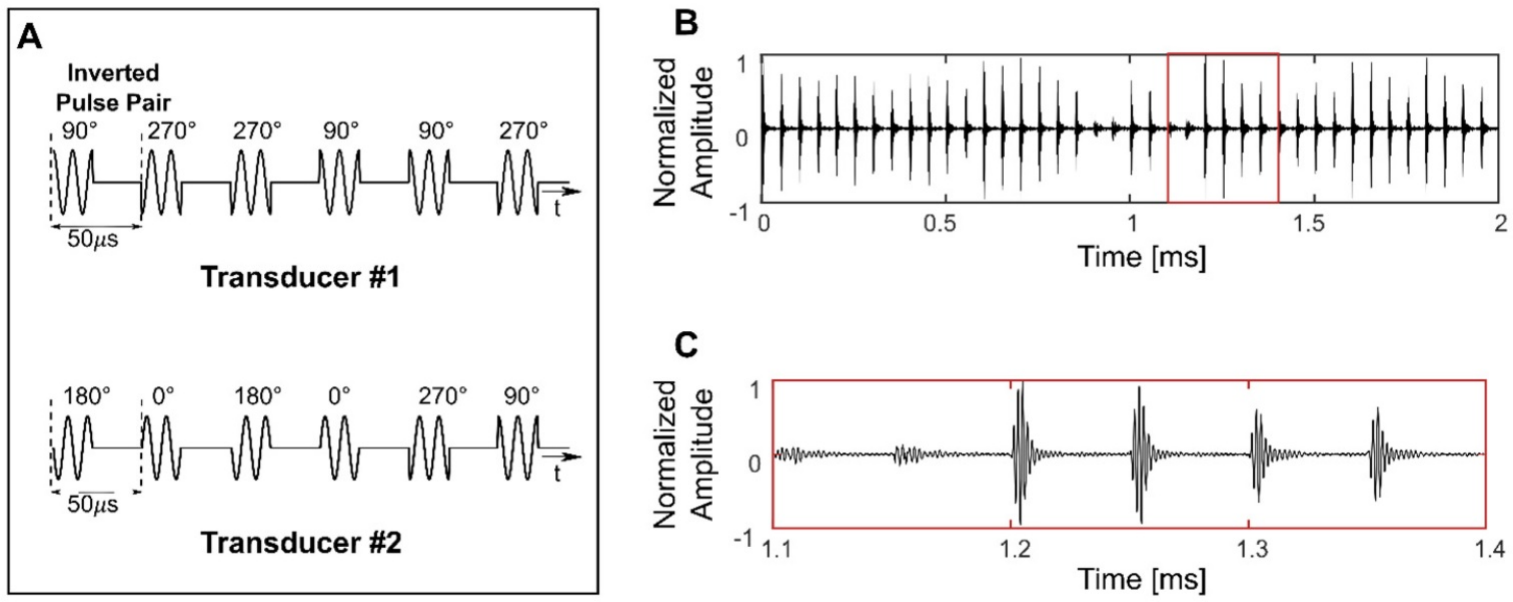
Short burst phase keying (SBPK) pulses generated in MATLAB (**A**) and, (**B**) and (**C**) measured at the focus using a polyvinylidene difluoride needle hydrophone. The waveform shown in (**C**) is a zoomed version of (**B**), indicated by the red box in (**B**). (**C**) shows the characteristics of SBPK exposures at the focus, including inverted pulse pairs and varying resultant magnitudes due to the interference of phase shifted pulses from the two transducers.

Both 10 ms sinusoidal bursts and SBPK exposures were investigated for BSCBO in this study. Prior to experiments, two SBPK pulse trains, one for each transducer were generated in MATLAB and uploaded to the dual-channel arbitrary function generator. Figure 2A shows segments of the pulses generated in MATLAB, while Figures 2B and C show waveforms measured on the benchtop at the focus of both transducers using the PVDF needle hydrophone.

### Focused Ultrasound Treatments

#### Animals

All animal experiments were approved by the Sunnybrook Research Institute animal care committee and were performed in keeping with guidelines from the Canadian Council on Animal Care. Eight (8) Yorkshire pigs (32.6 - 40.0 kg; Male; Lakeview Swine, Wallacetown, ON, Canada) were treated. To accommodate space constraints of the experimental set up, animals were requested from the supplier based on weight. Based on growth curves for Yorkshire pigs 61, the animals were estimated to be in the age range of 10-12 weeks at the time of treatment.

Animals were anaesthetized with ketamine (20 mg/kg, Narketan 10, Vetoquinol N.-A. Inc., Lavaltrie, QC, Canada) and atropine (0.02mg/kg, Teligent Canada, Mississauga, ON, Canada), intubated and maintained under anaesthesia using isoflurane (2%, Isoflurane USP, Fresenius Kabi Canada, Toronto, ON, Canada) and oxygen with medical ventilation. Prior to ultrasound exposures, the carrier gas was switched to medical air. To prevent an adverse reaction to the MBs 38-40, two (2) pigs were given intramuscular (IM) dexamethasone (0.5 mg/kg, Sandoz Canada, Boucherville, QC, Canada) twice a day for two days prior to the treatment. On treatment day, these pigs were administered an additional dose of dexamethasone (0.5 mg/kg). They were also administered a nonsteroidal, anti-inflammatory drug, meloxicam (0.4 mg/kg, Metacam, Boehringer Ingelheim Animal Health Canada Inc., Burlington, ON, Canada), for two days prior to the treatment and on treatment day. On treatment day, 30 min before the first treatment, intravascular (IV) diphenhydramine (0.5 mg/kg, Sandoz Canada, Boucherville, QC, Canada), an antihistamine, was administered. This drug cocktail was discontinued for the remaining six (6) animals and replaced with diphenhydramine (0.5 mg/kg) alone, administered in two half doses prior to the experiments. This change was made because the dexamethasone/meloxicam/diphenhydramine cocktail was found to restrict BSCBO. This is discussed further in the results and discussion sections. In animals receiving diphenhydramine alone, the first half dose was given IM after ketamine/atropine anaesthetization. The second half dose was diluted in 10 mL saline and given IV 30 min prior to the first treatment as a bolus injection. During experiments, heart rate and blood oxygenation levels were monitored in real time using a pulse oximeter (GE Datex-Ohmeda Pulse Oximeter- MRI Monitor, GE Healthcare, Chicago, IL, USA).

Following treatments, Evans blue dye (EBD) (4% weight per volume saline dilution, 2 mL/kg, Thermo Fisher Scientific, Waltham, MA, USA) was administered IV, as a bolus, and allowed to circulate for 1.5 h to assess BSCBO. In pigs 1-6, EBD was administered following a period of post treatment magnetic resonance imaging (MRI) (~1hr), while in pigs 7&8, EBD was administered immediately following the last treatment, prior to post treatment MRI. In pigs 7&8, a greater number of sonications were performed and the decision to administer EBD prior to the last treatment was made in an effort to keep the time between the first sonication and EBD administration to a minimum.

Spinal cords were formalin fixed through transcardial perfusion with saline followed by 10% neutral buffered formalin under deep anesthetic and harvested for histological staining. Posterior elements of vertebrae in the treatment region were removed and stored in formalin for use in benchtop experiments to estimate ultrasound transmission through the spine. In one (1) animal, three (3) whole vertebrae above and below the treatment region were also removed. The final four (4) pigs were given heparin (500 IU/kg, Heparin LEO, LEO Pharma Inc., Thornhill, ON, Canada) 30 min prior to perfusion to minimize blood clotting during perfusion.

#### Treatments

**Figure 3:**
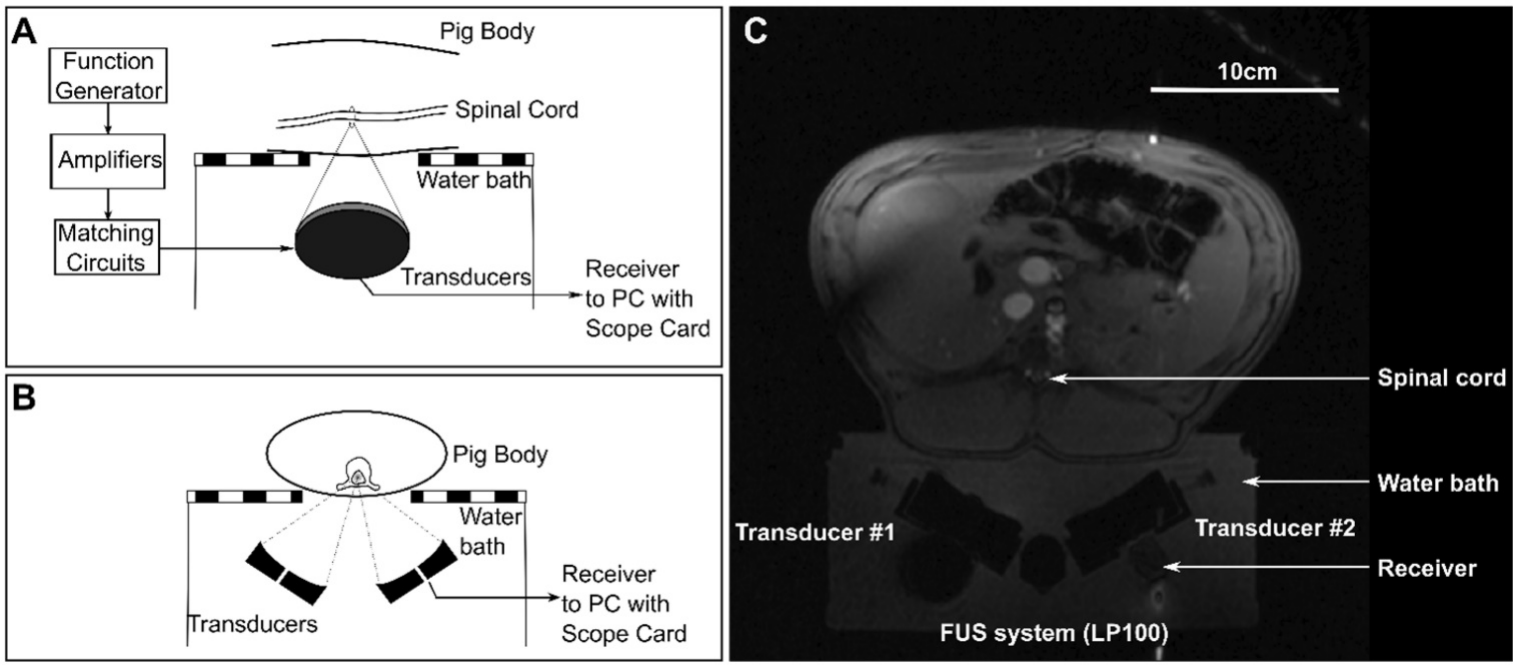
Schematic diagrams of (**A**) a sagittal view and (**B**) an axial view of the experimental set ups for treatments. (**C**) shows an axial magnetic resonance localizer image showing the experimental set up.

**Figure 4:**
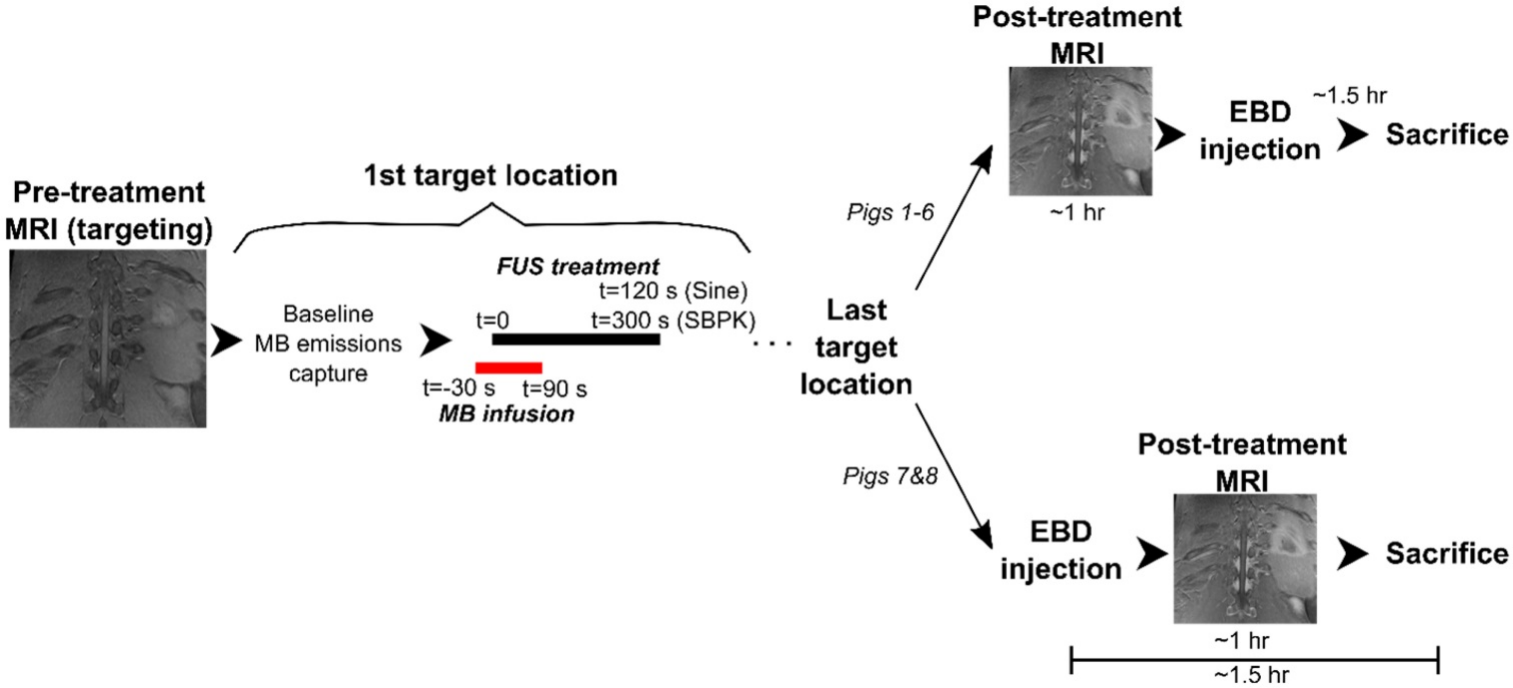
A schematic diagram showing a flowchart of the experimental procedure for each pig.

Animals were placed supine on top of a MRI compatible FUS system (LP-100, FUS Instruments, Toronto, ON, Canada) within the bore of a 3T MRI (3T Biograph mMR, Siemens Healthineers AG, Erlngen, Germany). The dual aperture transducer set up was mounted on a 3-axis positioning arm contained in the water bath of the FUS system. Treatment locations in the lower thoracic to upper lumbar region within the spinal cord were selected and targeted under MRI-guidance. The ribs were used as anatomical landmarks, with the highest treatment location between T11-T13. Diagrams showing the set up for treatments are shown in Figure 3 and a schematic of the treatment workflow is shown in Figure 4. A supine position of the animals minimized the effects of motion due to breathing. To stabilize the animals in this position, towel rolls were placed on either side of the pigs and the animals were tightly secured to the bed of the MRI. Pre- and post-treatment images were compared to confirm that movement of the animals had not occurred during the experiments. Targets within the spinal cord were selected using coronal T1 weighted MRI (GRE, Field of view = 20 × 20 cm^2^, spatial discretization = 0.4 mm, Slice thickness = 1.5 mm, Echo time = 2.46 s, Repetition time = 7 s, Rare factor = 1, Number of averages = 12). A flex coil placed across the chest of the animal above the FUS targeted region was used to improve the image signal-to-noise ratio (SNR). The experimental setup (animal supine on the FUS treatment platform) prevented placing the coil closer to the imaging region.

The treatment parameters for the eight (8) animals are shown in Table 1. Pigs 1-4 were pilot animals, used to demonstrate feasibility of BSCBO. These animals were treated using both sinusoidal bursts (10 ms, 1 Hz PRF, 2 min duration) and SBPK pulse trains (10 ms, 1 Hz PRF, 5 min duration). The non-derated pressures used for these animals were between 1 MPa and 4 MPa for sinusoidal bursts and between 1 MPa and 2 MPa for SBPK exposures. Once the potential for BSCBO had been established, four (4) animals (pigs 5-8) were treated at 2-10 locations per spinal cord, 10 mm apart, using ramped pressure SBPK exposures (10 ms pulse trains, 1 Hz PRF, 5 min). For these, pressures were ramped from 1.8 MPa to 2.1 MPa (non-derated) in 3 kPa increments over 2 min. A minimum of 8 treatment locations were intended in pigs 5-8. The varying number of sonications was a result of technical challenges encountered on the experiment days that limited the number of sonications performed in pigs 5 and 6.

For each treatment, MBs (0.005-0.010mL/kg, Definity, Lantheus Medical Imaging, MA, USA) were infused IV over 2 min. (The MB dose for pigs 1-5 was 0.010mL/kg. In pig 5, an increase in heart rate was observed following MB infusion. While vitals did return to normal, out of an abundance of caution, the MB dose was reduced to 0.005mL/kg for pigs 6-8.) FUS sonication commenced 30 s after the start of the infusion. A 250 kHz narrowband receiver was used to acquire acoustic emissions from MBs during treatments. Acquired waveforms were digitized using a two-channel 14-bit PCI scope card (ATS460, Alazar Technologies Inc., QC, Canada). For pigs 5-8, the frequency content of acoustic emissions, analyzed as described in 26, were plotted in real time and a detectable change in the subharmonic was used to trigger a 50% pressure drop, as described in 8. If a pressure change was triggered, the pressure was maintained at 50% of the trigger level for the duration of the treatment. If a pressure change was not triggered, the pressure was maintained at the maximum pressure (2.1 MPa) for the final 3 min of the treatment.

Following treatments, contrast enhanced (0.1mL/kg Gadovist (IV), Bayer Inc., NJ, USA) T1-weighted MR images were acquired using the parameters used for targeting, to assess BSCBO. For pig 8, additional coronal and sagittal contrast-enhanced, T1-weighted images (FLAIR, Field of view = 20 × 20 cm^2^, spatial discretization = 0.6 mm, Slice thickness = 1 mm, Echo time = 22 ms, Repetition time = 2 s, Inversion time = 860 ms, Rare factor = 5, Number of averages = 12) were acquired once all treatments were completed, removing the animal from the FUS treatment platform and imaging on the spine coils built into the MRI table. MR images were analyzed using Medical Image Processing, Analysis and Visualization (MIPAV; National Institutes of Health, Bethesda, MD, USA). BSCBO was confirmed if the mean MRI signal in a 3×3 voxel area centered on maximum enhancement was at least 2 standard deviations greater than the mean signal in a baseline 3×3 voxel unsonicated area in the spinal cord.

#### Histology

5 µm thick coronal sections at 100 µm intervals were stained using hematoxylin and eosin (H&E) and evaluated for tissue damage. Tissue damage at each treatment location was graded using a 4 point grading scheme adapted from 41. A description of each grade is shown in Table 2.

### Benchtop Experiments

#### Transmission Measurements

The posterior spinal segments that were removed after perfusions were used in benchtop experiments to estimate ultrasound transmission through the spine and update pressure estimates from the transducer calibration. The experimental setup used was similar to that used for transducer calibration (Figure 1B). Samples were degassed for several days in a vacuum jar, until no air bubbles were observed. On the day of the experiments, samples were degassed for 3 h in a vacuum jar prior to measurements. Posterior spinal (vertebral arch) samples from four (4) pigs (28.8-40.0kg) were placed between the transducers and the hydrophone. Field scans were performed with the interior surface of the vertebral arch positioned 6 mm pre-focal to the focal location as measured in water. Field scans were performed along three perpendicular planes with dimensions of 6 × 6 mm^2^. Vertebrae were translated along the sagittal plane (increments of 2.5 mm) to account for differences in transmission owing to vast inhomogeneities of the vertebral column.

#### CT Imaging

Although a one-to-one comparison of porcine and human vertebrae was not possible due to differences in the number of thoracic vertebrae between the two species, the sizes and densities of porcine vertebrae at/near the treatment region were compared with vertebrae in the human lower thoracic spine to estimate the representativeness of the porcine model used here.

One posterior vertebral segment (T12-T15) and three individual vertebrae (T9-T11) were degassed for several days in a vacuum jar, until no air bubbles were observed. On the day of the imaging, samples were degassed for 3 h before imaging using an x-ray computed tomography (CT) scanner (Aquilon One, Toshiba Tec., Markham, ON, Canada). The vertebrae were aligned in the scanner, such that an axial plane across the vertebrae would be aligned with the axial plane of the scanner (i.e. as if they were in the body). Vertebrae were imaged with a resolution of 0.5 mm, so that they would be comparable to existing CT scans of *ex vivo* lower human thoracic vertebrae (T7-T12) previously used for bench and computational studies by our group 31.

The vertebrae were segmented using semi-automatic segmentation in ITK-SNAP 42 and 3D renderings of segmented data were visualized in MeshLab 43. CT scans and segmentation masks were imported into MATLAB to analyze vertebral density. As some tissue was left on the excised porcine vertebrae, the authors were concerned that some of this soft tissue would affect CT density analysis. Therefore, the top 5% of CT intensity voxels were considered to strictly isolate bone and to disregard water, air or soft tissue that may have been missed during segmentation.

### Thermal Simulations

To confirm that observed tissue effects, like BSCBO, were mechanical rather than thermal, 2D simulations were performed in k-Wave 44. The *pstdElastic2D* function was used to compute the elastic propagation of SBPK exposures (10 ms, 486 kHz, fixed pressure), in the dual aperture configuration, through the vertebral laminae (porcine T11) to the spinal canal. Medium densities were assigned using CT derived vertebral densities. Compressional and shear speeds of sound and attenuation coefficients were extrapolated from experimental data presented in 33 and 45. Following the acoustic simulations, the volume rate of heat deposition at each location in the simulation space was calculated based on the resultant pressures recorded in the time domain 46. This was used as input for *kWaveDiffusion* to compute a solution to Pennes' bioheat equation 47. Values of thermal conductivity and specific heat capacity were taken from 46, and absorption in the spinal cord was assumed to be the same as in the brain (5 Npm^-1^MHz^-1^ 4). Thermal simulations were conducted over 5 min, with 10 ms heating every 1 s, to replicate the treatment scenario.

## Results

### Blood spinal cord barrier opening (BSCBO)

**Figure 5:**
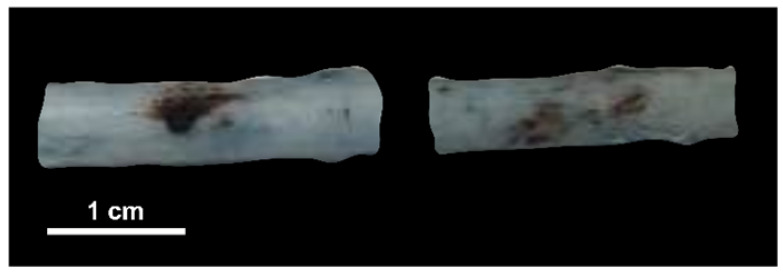
Macroscopic damage (haemorrhage) observed without blood spinal cord barrier opening in pigs given dexamethasone and meloxicam (pig 2).

In pigs 1 and 2, the cocktail of dexamethasone and meloxicam was found to restrict BSCBO. At locations treated with pulsed, sinusoidal exposures, hemorrhage was observed without EBD, as shown in Figure 5. This indicates that the BSCB was not substantially modified prior to the threshold for damage, or that the closure time following opening was very short and the barrier was no longer open at the time of EBD injection. These animals were excluded from further analysis and the remaining animals were only given diphenhydramine. These findings are discussed further in comparison with existing literature in the brain in the discussion section.

**Figure 6:**
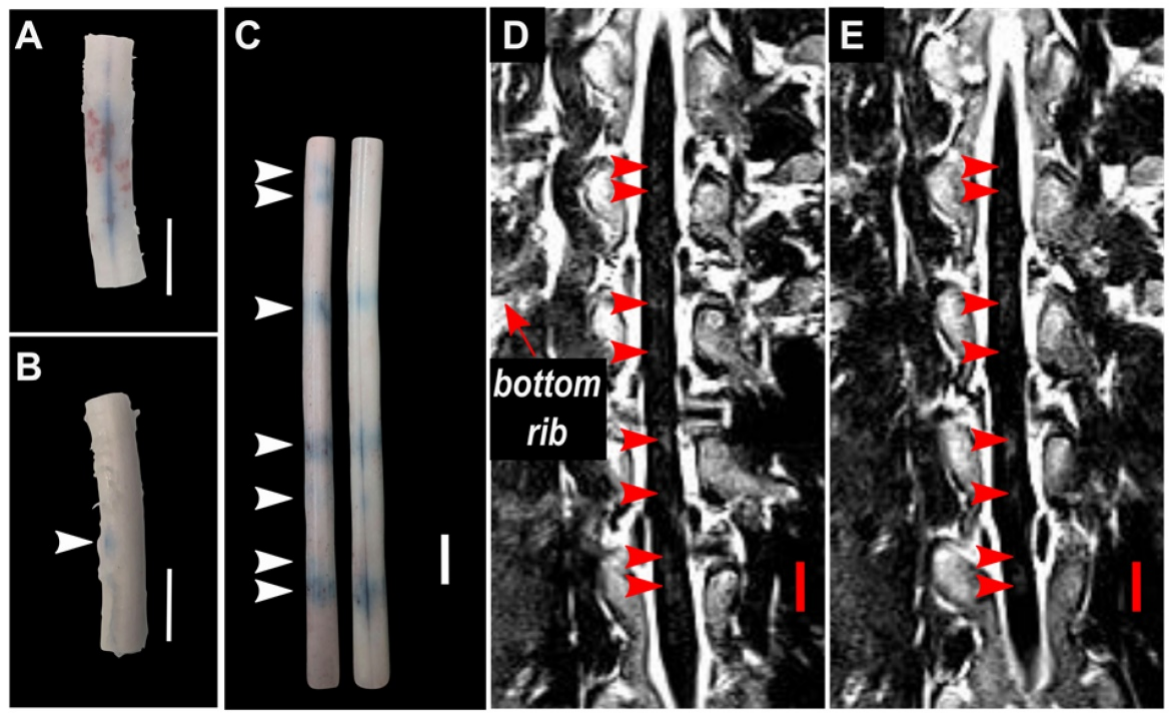
Blood spinal cord barrier opening using (**A**) pulsed sinusoidal exposures showing haemorrhage (pig 4) and (**B**) SBPK exposures (pig 4) confirmed by Evans blue dye (EBD) extravasation. (**C,D,E**) Blood spinal cord barrier opening in pig 8 confirmed by (**C**) EBD extravasation (left: posterior view, right: anterior view) and (**D,E**) adjacent coronal slices of post-treatment contrast enhanced T1-weighed MRI with the bottom rib labelled as an anatomical landmark. In (**B**) and (**C**), the arrows represent locations where EBD was observed. In (**D**) and (**E**), the arrows represent the locations at which gadolinium contrast enhancement was observed in any image slice (n=12). All scale bars are 1cm.

A summary of BSCBO and observed macroscopic damage at all treatment locations in pigs 3-8 is shown in Table 3. In pigs 3 and 4, EBD extravasation was observed for all treatments using 10 ms sinusoidal bursts. However, this was accompanied by extensive macroscopic damage, defined as observable hemorrhage on the surface of the cord. BSCBO observed using sinusoidal exposures in pig 4 is shown in Figure 6A. Nevertheless, this indicated that sufficient FUS could be delivered, through the intact spine, to achieve a therapeutic effect. In pig 4, EBD extravasation was also observed at one (1) location treated using SBPK exposures, which is indicated in Figure 6B. Here, BSCBO appeared more localized and there was no macroscopic damage observed. No effects were observed on contrast enhanced, T1-weighted GRE MRI. Pigs 3 and 4 were pilot animals, and were treated before a more robust and repeatable treatment protocol was established for pigs 5-8. In these animals, the treatments were very close together, sometimes overlapping, and consisted of multiple point sonications, making it difficult to distinguish EBD extravasation from different treatments. Therefore, the EBD extravasation reported in Table 3 for these animals is not location specific, and combines effects observed for each pulse type.

In pigs 5-8, which were treated with ramped pressure SBPK exposures, EBD extravasation was observed at 16/24 targeted locations. Some macroscopic damage was observed at three (3) of the locations with successful BSCBO in pig 8. An example from pig 8 is shown in Figure 6C, where 7/10 treatment locations had EBD extravasation. In this pig, although seven (7) BSCBO locations were confirmed using EBD extravasation, eight (8) locations were confirmed using post treatment contrast-enhanced T1-weighted FLAIR MRI (Figure 6D, E). The mean enhancement resulting from BSCBO in this animal was (31.8 ± 8.5 %) (21.7% - 48.9%) [(mean ± standard deviation) (minimum-maximum)].

**Figure 7:**
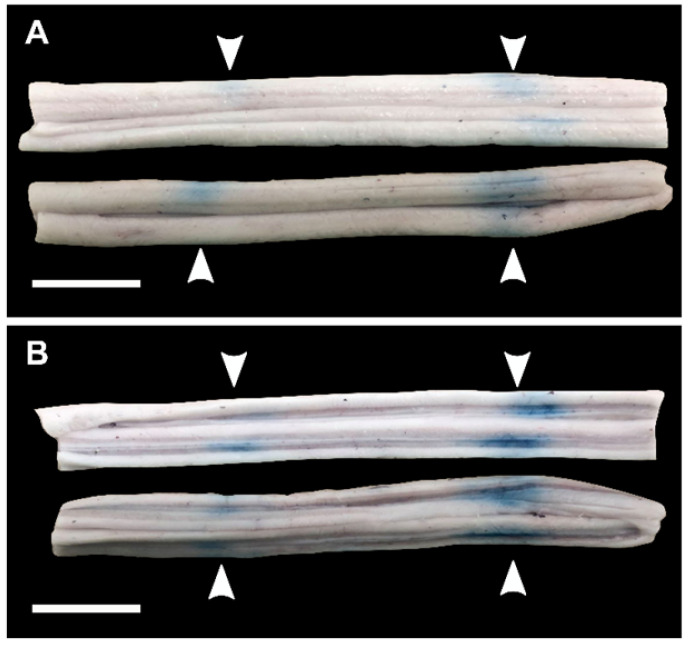
Blood spinal cord barrier opening confirmed by Evans blue dye (EBD) extravasation in bisected spinal cord segments in one animal (pig 7). (**A**) shows the exterior of the cords (white matter) and (**B**) shows the interior of the cords (grey matter). The white arrows indicate the locations at which EBD extravasation was observed. All scale bars are 1cm.

In two (2) animals, pigs 5&6, spinal cords were bisected coronally. Qualitatively, the results showed more intense EBD extravasation in the grey matter of the spinal cord compared with the white matter (Figure 7).

### Histology

**Figure 8:**
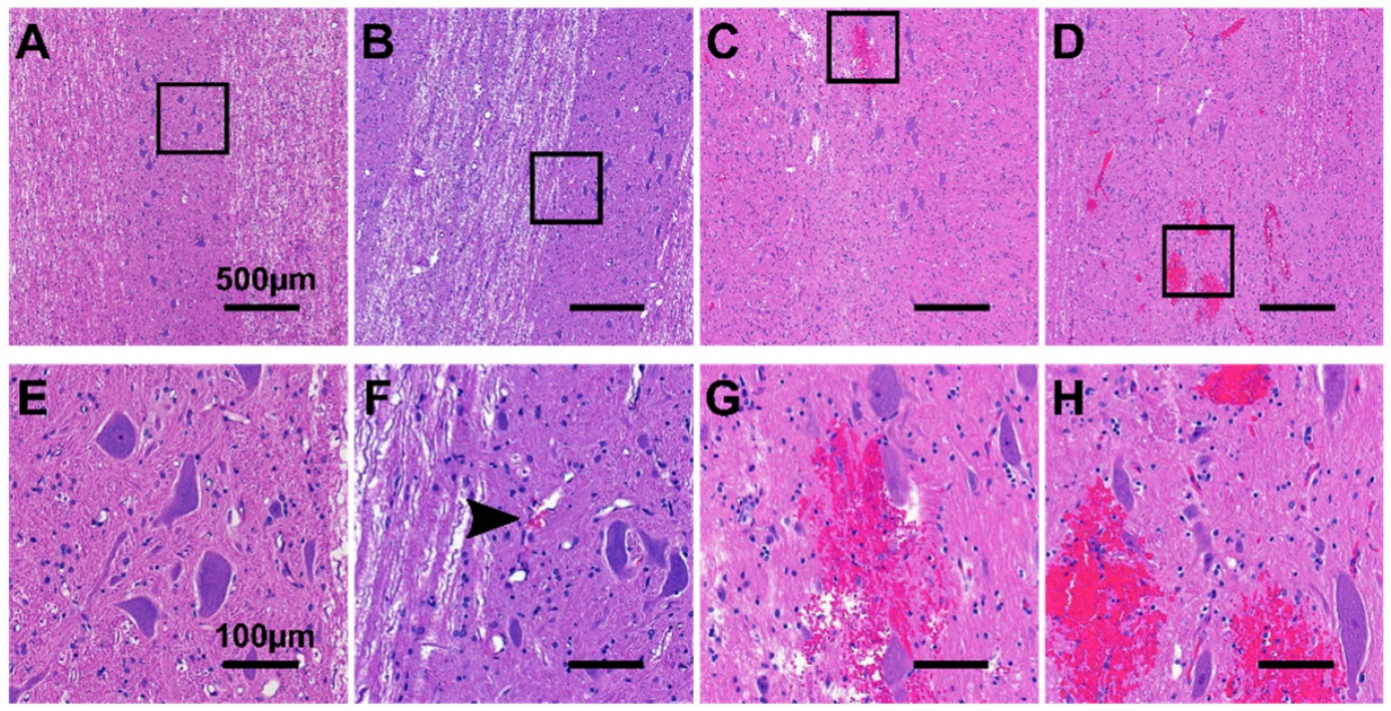
Representative H&E stained histology images showing (**A**,**E**) grade 0 damage, (**B**,**F**) grade 1 damage, (**C**,**G**) grade 2 damage, and (**D**,**H**) grade 3 damage. (**E**-**H**) are 5x magnifications of (**A**-**B**). The black arrow in (**F**) indicates a small cluster of extravasated red blood cells.

Figure 8 shows representative histology examples of H&E stained histology images for the four histology grades: grade 0 (A&E), grade 1 (B&F), grade 2 (C&G), and grade 3 (D&H). Damage was more prominent in the grey matter compared with the white matter, with the exception of animals treated with 10 ms sinusoidal exposures where damage was widespread throughout the cord.

The spinal cords of pigs 6 and 7, which were bisected, were excluded from histological scoring. Due to the position of the cut, tissue was lost in the grey matter region, making it difficult to draw conclusions after processing.

A summary of histology scores for each treatment is shown in Table 3. The scores presented in Table 3 represent the maximum value across histology levels (n=16) for each spinal cord.

### Acoustic emissions analysis

Using ramped pressure exposures, during treatments, a change in the pressure based on a detected subharmonic was never achieved. The method of Fourier analysis used in real-time was described in 26. There was one exception, at location 2 in pig 7, where a pressure drop was triggered at 46 s due to a false positive arising from a signal disruption during baseline measurements.

However, post-hoc analysis of the acoustic data showed that performing a subtraction of baseline data from treatment data in the time domain, before Fourier analysis, rather than performing a magnitude subtraction in the frequency domain, allowed for detection of the subharmonic (243 kHz), in most cases the 2^nd^ harmonic (972 kHz) and in some cases the 1^st^ ultraharmonic (729 kHz) using the 250 kHz PZT receiver. Acoustic data were available for pigs 4-8, with the exception of location 1 in pig 7, where the receiver experienced a technical failure.

**Figure 9:**
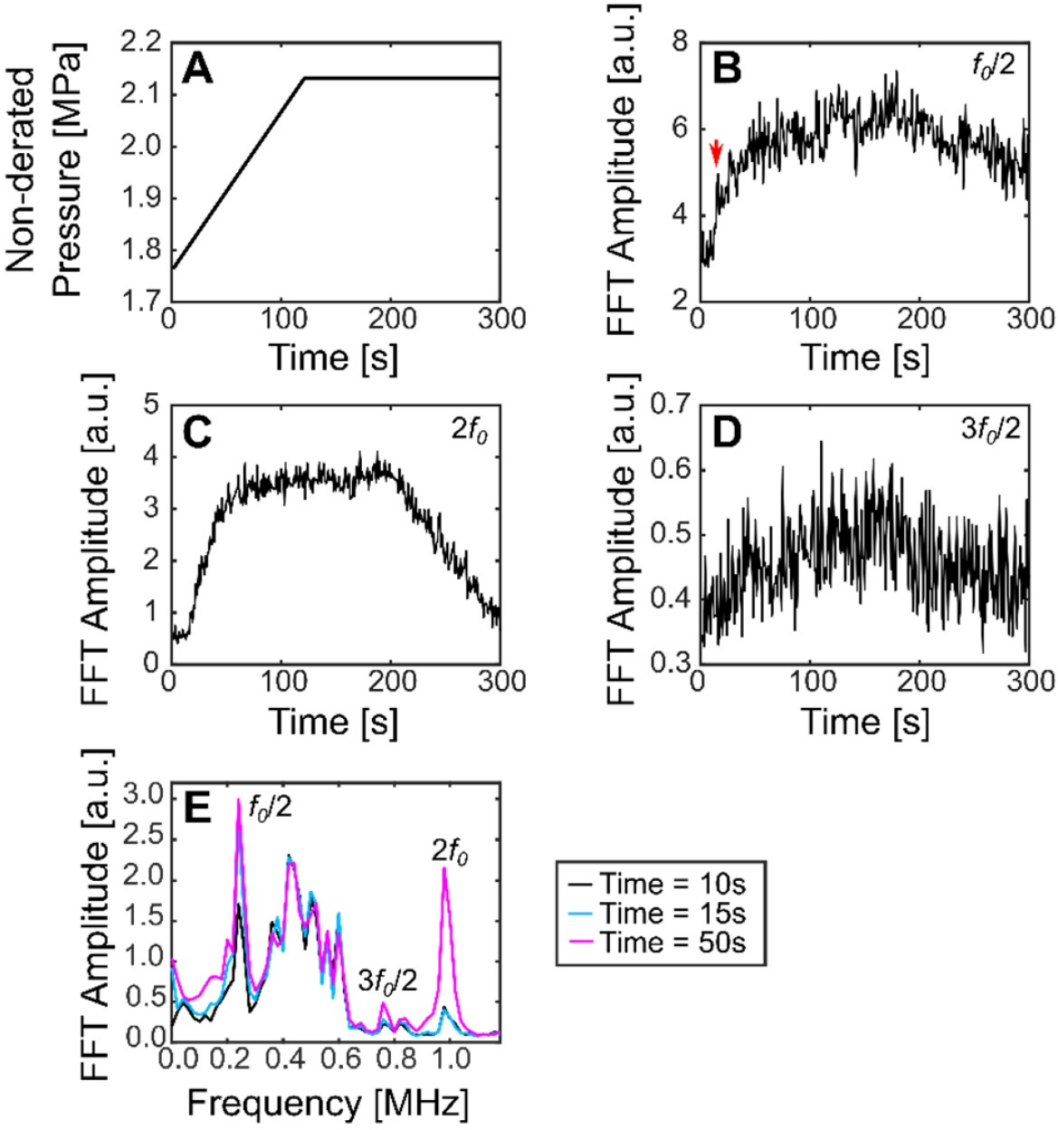
Plots showing (**A**) non-derated pressures during ramped SBPK sonication, (**B**) changes in the FFT amplitude at the subharmonic (a jump in the subharmonic at 15 s is indicated by the red arrow), (**C**) changes in the FFT amplitude at the 2^nd^ harmonic, and (**D**) changes in the FFT amplitude at the ultraharmonic. (**E**) shows maximum intensity projection frequency spectra at relevant time points. (**B**-**E**) correspond to location 2 in pig 8.

Using this method, the subharmonic was detected for all treatments in pig 4 (sinusoidal and SBPK exposures). For ramped exposures, a change in the subharmonic was defined as an increase in the summation over a 40 kHz band centered on the frequency of interest of more than five (5) standard deviations above the mean of the first 10 sonications. This metric was chosen over two (2) standard deviations as it was determined empirically to decrease the likelihood detecting outliers in the data. Using two (2) standard deviations, false positives were detected in 39% of treatments. Changes in the subharmonic were observed for 20/23 locations treated with ramped exposures and viable acoustic data. These changes were observed in the range 1.9-2.1 MPa (non-derated), between 15 s and 143 s. Interestingly, the subharmonic was detected at four (4) locations where BSCBO was not confirmed. Figure 9 shows the pressure ramp (A) and changes in the subharmonic (B), the 2^nd^ harmonic (C), and the 1^st^ ultraharmonic (D). Figure 9E shows the maximum intensity frequency spectra at relevant time points: before subharmonic detection (10 s), initial detection of subharmonic (15 s), and a time point well after the detection of the subharmonic (50 s). A summary of the detection of the subharmonic at all treatment locations with available acoustic data is shown in Table 3.

### Benchtop experiments

#### FUS transmission through bone

**Figure 10:**
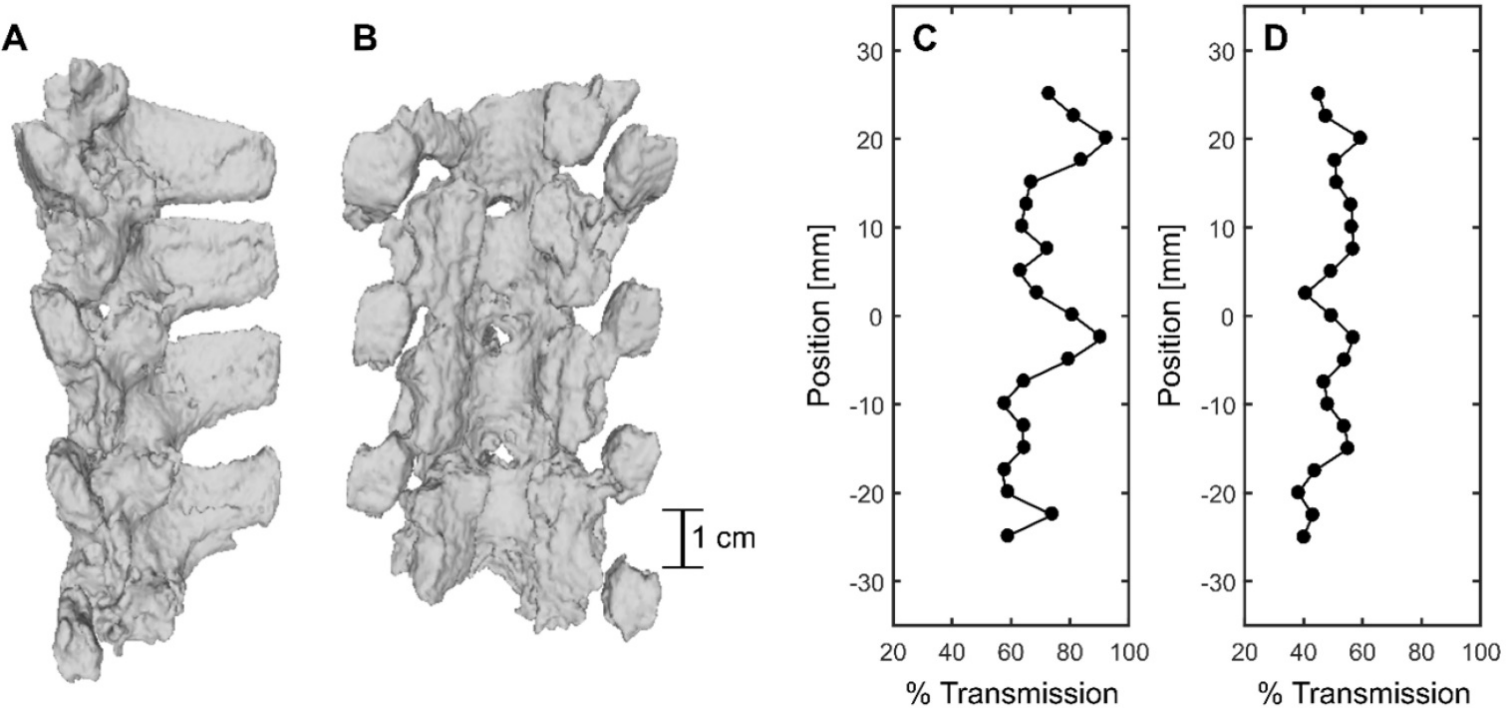
CT-derived 3D renderings of (**A**) the sagittal view and (**B**) the coronal view of the posterior elements of the porcine vertebral column. (**C**) and (**D**) show % transmission within a 6mmx6mm field of view and at the focus location compared with a water case, respectively.

CT-derived 3D renderings of the posterior elements of the porcine vertebral column in the lower thoracic region are shown in Figure 10A and B. Benchtop experiments through the posterior of four (4) vertebrae in the sonicated region at 69 locations, across four (4) spine samples, indicated a mean transmission to the target of (47.3 ± 7.0 %) (31.7%-61.9%) [(mean ± standard deviation) (minimum-maximum)]. However, transmission within a 6 × 6 mm^2^ field of view in three (3) perpendicular planes was (67.6 ± 12.1 %) (48.5%-95.0%). The mean focal shift was (2.7 ± 0.7) mm (0.8 mm - 3.0 mm). Examples of transmission to the target and within the 6 × 6 mm^2^ field of view are shown in Figure 10D and C respectively. The results show that % transmission is highly location dependent, with close to 100% transmission within a 6 × 6 mm^2^ when targeting near an acoustic window. It is likely that pressures could be further derated due to intervening muscle and additional connective tissues not taken into account in these measurements. Additionally, in the experimental set up, pigs were slightly in extension due to lying supine, so acoustic windows may have been more narrow than in the benchtop experiments.

#### Comparison of vertebral geometry in pigs and humans

**Figure 11:**
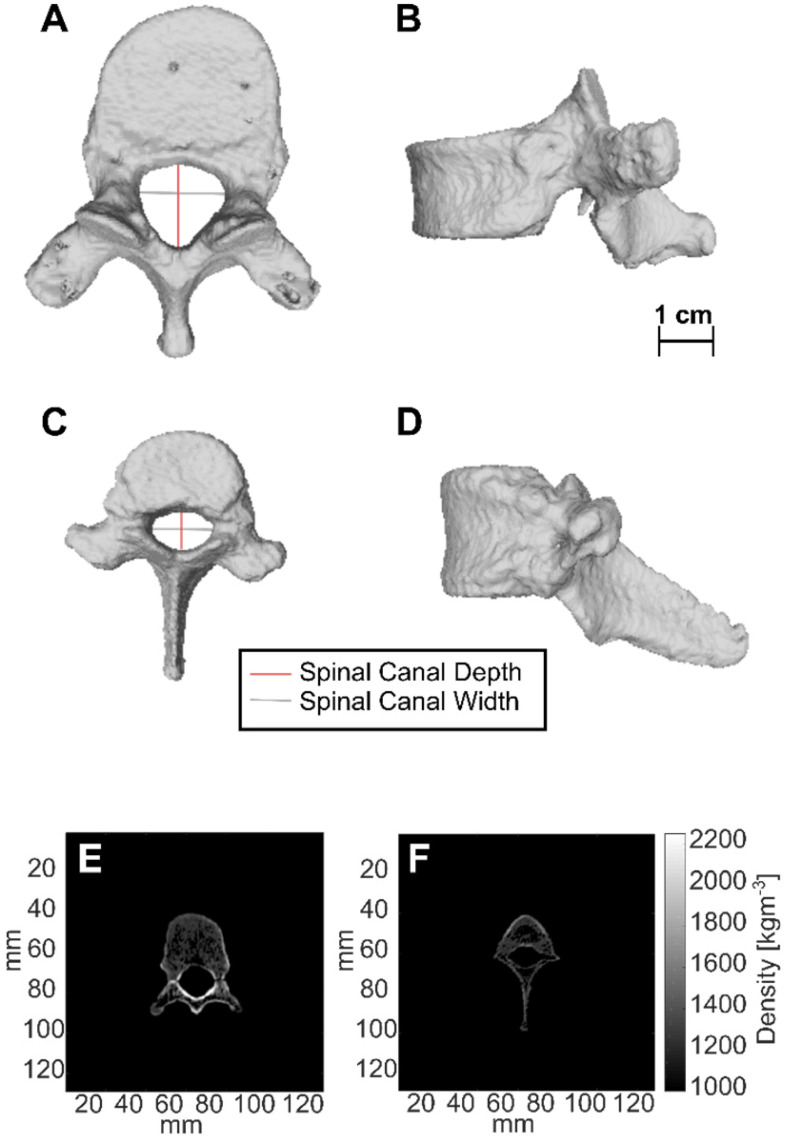
CT-derived 3D renderings of (**A**) the top view and (**B**) the side view of the 10^th^ human thoracic vertebra (T10), and CT-derived 3D renderings of (**C**) the top view and (**D**) the side view of the 11^th^ porcine thoracic vertebra. (**E**) and (**F**) are axial slices showing the CT derived densities of the 10^th^ human thoracic vertebra and the 11^th^ porcine thoracic vertebra respectively.

CT-derived 3D renderings of the 10^th^ human thoracic vertebra (T10) and the 11^th^ porcine thoracic vertebra (T11) are shown in Figure 11. Measurements of spinal canal depth and width measured in porcine samples and human samples are shown in Table 4. On average, the spinal canal depths of the measured porcine vertebrae were (58 ± 4 %) of the spinal canal depths measured in the lower thoracic region of the human spine, while the spinal canal widths were (85 ± 6 %) of the spinal canal widths measured in the lower thoracic region of the human spine. The errors in Table 4 are the standard deviations of measurements across 20-38 CT slices.

The values of the CT derived density for the top 5% of voxels in each vertebra after segmentation are shown in Table 5. These values were computed separately for the vertebral bodies and for the posterior elements of the vertebrae. In the vertebral bodies of the lower thoracic spine, the porcine densities were on average (90 ± 4 %) of the human densities. In the posterior elements of the lower thoracic spine, the porcine densities were on average (70 ± 2 %) of the human densities.

### Thermal Simulations

Simulation results showed that at the maximum pressure used for ramped SBPK exposures, 2.1 MPa, the corresponding temperature rise in the spinal canal would be in the range 0.07 °C - 0.33 °C (assuming transmission through vertebrae of 48.5% - 95.0%). With MBs present, increased heating by a factor of ~3.6 can be expected 48. This corresponds to a temperature rise between 0.25 °C and 1.19 °C over the 5 min treatment duration. These temperatures are unlikely to result in thermally induced blood-spinal cord barrier opening 49.

## Discussion

In this body of work, we have presented a porcine model of BSCBO through the intact spine, using SBPK FUS + MBs. Successful modification of the BSCB, as confirmed by the extravasation of EBD, and benchtop measurements of transmission through the vertebral column, indicate that minimally invasive (i.e. without laminectomy) delivery of FUS to the spinal cord is feasible.

BSCBO was confirmed by the extravasation of EBD using 10 ms sinusoidal pulses and 10 ms SBPK pulse trains. This study was not designed to evaluate the differences between these two pulse types. The treatment pressures used for sinusoidal (1.0 - 4.0 MPa) and SBPK (1.8 - 2.1 MPa) exposures, and the treatment protocol used were not directly comparable, but relevant observations are discussed here. While it is difficult to comment on levels of opening and damage, due to differences in pressure, there were differences observed in the distribution of opening and damage throughout the cord with the different pulse types. This is especially relevant in pig 4, where both pulse types were used with the same target spacing (Figure 6 A&B). Using sinusoidal exposures, widespread hemorrhage was observed on the surface of the cord in the treatment region. Upon histological scoring, extensive (grade 3) damage was observed throughout the cord on all histology levels. SBPK exposures resulted in BSCBO that was qualitatively more localized than long burst exposures. Further, hemorrhage on the surface of the cord was not observed in most cases. In the cases (3/24 locations treated with ramped SBPK exposures) where macroscopic damage was observed, it was qualitatively less prominent than in the sinusoidal cases. At locations treated with SBPK FUS, the most substantial damage observed through histological scoring was often observed at the center of the cord in the grey matter. The authors hypothesize that these differences could be explained, at least in part, by the differences in focal uniformity between the pulse types in the dual aperture, cross-beam configuration and the reduced potential for the formation of standing waves using SBPK pulse trains that was observed in a study using *ex vivo* human vertebrae 35. However, further experimental confirmation is required.

Another observation of this study was that when BSCBO was achieved, EBD extravasation at the center of the cord in the grey matter was more prominent than in the white matter. This effect has previously been reported in a study of BSCBO in the rabbit spinal cord using unfocused ultrasound, following laminectomies 27, and also in the brain in non-human primates 11. Although the spinal cords of two (2) animals were sectioned to assess this effect, sectioning was found to compromise histological processing and scoring, so it was not done for other animals. It is therefore possible, that treatment effects confined to the grey matter of the cord could be missed when assessing using EBD extravasation. In fact, in pig 8, where MRI enhancement was observed and quantified, there was one treatment location at which BSCBO was confirmed by MRI (enhancement = 24%), but not by EBD extravasation. This could mean that opening was localized in the grey matter. The differences observed throughout this study between effects observed in the grey and white matter of the spinal cord are likely due to differences in vascularization. The grey matter is supplied by a dense network of capillaries, while the white matter is poorly supplied 50. An additional explanation for the location in pig 8 that showed BSCBO on MRI but not by EBD extravasation is the comparative size of the tracers. Gadovist, the MRI contrast agent used, has a molecular weight of 604.7 Da 51, while serum albumin, to which EBD binds, has a molecular weight of 66.5 kDa 52. This implies that EBD could be less sensitive to the detection of limited BSCBO compared with contrast enhanced T1-weighted MRI, which is a limitation of the results presented here.

Differences in the time between treatment and administration of tracers (EBD and gadolinium) were not taken into account in animals that received multiple treatments. Because these differences could affect the results observing BSCBO, quantitative comparisons of opening are not presented here. The maximum time between the FUS treatment and EBD administration was ~5 hr (pig 8, location 1). For the last treated location, this time was ~1 hr (pigs 1-6) or immediate (pigs 7&8), depending on whether post treatment MRI was performed before or after EBD administration. Studies in the brain indicate that increased permeability can persist for several hours to days after the treatment depending on factors, including the magnitude of initial opening 4,53,54. This is a further limitation of this study, because, for animals with many target locations, minimal opening occurring at one of the early target locations may have been missed using the current methods.

Definity MBs used during treatments are lipid encapsulated microspheres. Early in the study, in order to minimize the potential for an allergic reaction to liposomes that has been reported in pigs 38-40, animals were given a cocktail of dexamethasone, meloxicam and diphenhydramine. Prior research in the rat brain has shown that post-treatment administration of dexamethasone can help reduce the permeability of the blood-brain barrier after FUS-induced barrier opening and reduce the risk for tissue damage resulting from inflammation 55. In that study, rats were administered dexamethasone or saline 35 min and 24 h post FUS treatments. Using dynamic, contrast enhanced MRI, the reduction in permeability of the barrier at the 2 h time point compared with the 15 min time point was shown to be greater in the dexamethasone group than in the saline group. In our study, the spinal cords of animals that were administered dexamethasone prior to the treatment had haemorrhage without the observable extravasation of EBD. We hypothesize two possible explanations. Firstly, since dexamethasone was administered before FUS treatments, it may have preserved the integrity of the BSCB, such that opening was not achieved before the threshold for damage was reached. Alternately, due to the time required for treatment of multiple locations and post-treatment imaging, the time between FUS delivery to the final treatment location and EBD administration was >1 h in the animals who received dexamethasone. This could mean that if BSCBO was achieved, dexamethasone may have led to expedited restoration of the barrier in the time between treatment and EBD administration.

During treatments, attempts to leverage the acoustic emissions to control treatment exposures were unsuccessful. In post-treatment analysis of received acoustic signals, changes at the 2^nd^ harmonic, subharmonic and 1^st^ ultraharmonic frequencies were observed. However, changes at the frequency of interest, the subharmonic, were observed at the majority of locations treated, even at locations at which BSCBO was not confirmed. There were three (3) locations where the subharmonic was not observed. BSCBO was also not observed at these locations. These results could be explained by the following reasons. Firstly, the pressures used in this study are much higher than those used in previous studies of BSCBO 24,26. This statement is true even when considering maximum transmission losses through the porcine spine measured in benchtop experiments. It is possible that, because of this overexposure, the detected subharmonic signals are indeed indicative of bubble events in the porcine spinal cord, and should have resulted in a drop in pressure using the controller. Alternatively, considering the limitations of a set up using a single acoustic receiver, the received acoustic signals cannot be localized to events in the spinal canal. *Ex vivo* experimental and simulation work from our lab suggests the potential for pre-focal effects resulting from reflections off the vertebral laminae, potentially with acoustic pressures that are higher than those achieved within the spinal canal 32. This explanation is supported by the observation of increased delivery of EBD in intervening tissue in some animals in this study. A better picture of bubble effects within the spinal cord could be provided by using large aperture phased arrays and passive acoustic mapping for receiving bubble signals, as has been done in brain studies 56-58. Additionally, using a receiver with a central band that is not at the frequency of interest or a broadband receiver, could be useful for better detecting changes in acoustic signals.

Using SBPK FUS exposures, there were four (4) treatment locations at which BSCBO was achieved with very minor damage (grade 1) observed on histology. Inefficiencies in the transcardial perfusion technique at animal sacrifice meant that red blood cells were observed within vessels throughout the spinal cords, even outside of the treated regions. Depending on the orientation of blood vessels relative to the orientation of cutting for histology, it was difficult to distinguish intravascular clusters of red blood cells from grade 1 damage. Although grade 1 damage has been reported, inefficiencies in the transcardial perfusion technique may have led to the possible misclassification of grade 0 tissue. The understanding of tissue damage obtained in this study is also limited by the acute timepoint at which transcardial perfusion was done. It is possible that damage at locations graded 1 or 2 could be resolved at later timepoints as indicated by studies in the brain 8. This is an important result as it indicates that FUS-induced BSCBO may be achieved with minimal adverse effects for spinal cord tissue.

Benchtop testing of vertebral samples taken from animals after treatments revealed differences in vertebral size and density of pigs compared with humans. This analysis is limited as it compares one porcine spine with one human spine. Another limitation of this analysis is that it was performed *ex vivo*. While *in vivo* CT measurements would have been more relevant to the clinical case, *ex vivo* CT of human vertebrae and transmission measurements were readily available and this allowed for a more direct comparison. In the lower thoracic to upper lumbar region, pigs had a larger spinal canal width to depth ratio, with a spinal canal width of (58 ± 4)% and a spinal canal depth of (85 ± 6)% of samples in the human lower thoracic region. This geometric difference will affect how waves due to reflections within the vertebral canal interfere with each other. This decrease in symmetry could result in reduced potential for standing waves compared with humans. However, the smaller target within the porcine canal could lead to the opposite effect.

While the CT derived densities of porcine vertebral bodies in the lower thoracic region were (90 ± 4 %) of those in humans, densities in the posterior elements, including the laminae were (70 ± 2 %) of those in humans. This was an unexpected result, as vertebral densities in quadrupeds have been reported to be higher than humans, at least in the vertebral bodies 59. This discrepancy is most likely due to limitations placed on the weight of the pigs used in this study wherein in order to fit the animal and treatment platform within the MRI bore, they had not achieved maturation at the time of treatment. It is known that bone mineral density in growing animals' increases and reaches a peak at the point of maturation 60. The difference in bone geometry and density likely explains the higher levels of transmission through the pig vertebral laminae compared with humans that was observed. Over four (4) pigs, the mean transmission to the target measured on the bench was (47.3 ± 7.0 %) compared with a transmission of (31 ± 17 %) in humans 32 and (67 ± 15 %) in rats 24 at similar ultrasound frequencies. The authors hypothesize that the variability in transmission could be responsible for not observing BSCBO at some locations. Benchtop experiments also revealed the potential for pressure maxima occurring away from the intended focus, with a mean focal shift of (2.7 ± 0.7) mm (0.8 mm - 3.0 mm). This provides a possible explanation for the observed lack of uniformity in spacing of regions of EBD extravasation in some cases (Figure 6C).

In this study, control animals, without administered MBs, were not investigated. This control group would have been useful to distinguish whether the observed effects were due to low-power mechanical effects 4 or a high-power effect, like hyperthermia 49. However, the results of thermal simulations at the maximum pressures used (maximum temperature rise = 1.19 °C) and the low power levels associated with sparsely distributed SBPK exposures (maximum spatial peak temporal average intensity = 0.05 Wcm^-2^) provide convincing evidence that the thresholds for achieving thermally induced BSCBO would not have been met 49.

There are several limitations of this study and a range of challenges were encountered that should be addressed in future studies. Additional studies refining the treatment approach and examining chronic effects, including the impact on locomotor function, are needed. The authors propose several recommendations for future investigations of BSCBO in pigs or other large animals. Firstly, the inability to detect MRI enhancement, with the animal on the FUS system, using GRE sequences used to assess BBB opening was a major setback of this study. While the use of a FLAIR sequence together with placing animals on a spine coil gave better results, a spine coil that is compatible with the treatment system needs to be developed to permit accurate MRI assessment of opening during treatments. The development and implementation of a FUS-compatible spine coil will permit more robust and extensive MRI, including contrast-enhanced T1, T2 and T2* imaging to assess both BSCBO and toxicity. Secondly, a working controller to promote BSCBO while minimizing tissue damage is needed. In addition to the changes made in post-treatment analysis for this study, additional benchtop work is needed to develop an acoustic emissions based controller that is specialized for the application of SBPK pulses in the spinal cord. Greater receiver sensitivity is needed, and a receiver array to localize bubble events occurring in the spinal cord that offers the potential for passive acoustic mapping would also be advantageous. Finally, as evidenced by benchtop experiments, ultrasound transmission through the spine is location dependant and results in focal shifts on the order of several millimeters. This result reiterates the necessity for a transmit phased array to account for aberration as ultrasound transverses different paths through bone before arriving at the target within the spinal canal. A spine-specific phased array to address this concern is currently under development 31. A multi-element, phased transmit array would also allow for rapid electronic steering of the focus for treatment of diffuse disease.

## Conclusion

This study presents the first evidence of FUS-induced BSCBO in a clinically relevant, large animal model through the intact spine. FUS was delivered using SBPK, which is a pulse scheme designed for use in the human spine to reduce standing waves and focal depth of field in a dual aperture configuration. Using SBPK exposures, at several locations BSCBO was observed with minimal tissue damage observed through histology, demonstrating the potential of this approach. During ramped pressure SBPK exposures, changes in acoustic signals from MBs were observed, although it is unclear whether these could be attributed to MBs within the spinal canal or in the intervening soft tissue. Future studies are needed to refine the treatment approach and assess chronic treatment effects. Despite the challenges and limitations of this study, this work represents an important step towards clinical translation of non-invasive FUS-induced BSCBO.

## Figures and Tables

**Table 1:** Treatment parameters used during experiments

Pig ID	Pulse Type	Number of Treatments	Location Spacing (mm)	Pressure	Notes
1	SBPK	3		1. Fixed (1.5 MPa)	Dexamethasone and meloxicam
				2. Fixed (2.0 MPa)	4 point square grids (3mmx3mm)
				3. Fixed (1.0 MPa)	3mm spacing
2	Sine	2		1. Fixed (2.0 MPa)	Dexamethasone and meloxicam
				2. Fixed (4.0 MPa)	3-5 points per treatment, 4mm spacing
	SBPK	1		1. Fixed (2.0 MPa)	
3	Sine	4		1. Fixed (1.5 MPa)	4 point square grids (3mmx3mm)
				2. Fixed (2.0 MPa)	3mm spacing
				3. Fixed (2.0 MPa)	All points overlapping
				4. Fixed (3.0 MPa)	
4	Sine	2		1. Fixed (1.0 MPa)	3 points per treatment, 5mm spacing
				2. Fixed (4.0 MPa)	
	SBPK	1		1. Fixed (2.0 MPa)	
5	SBPK	2	10	Ramp (1.8-2.1 MPa)	Single point sonications
6	SBPK	4	10	Ramp (1.8-2.1 MPa)	Single point sonications
7	SBPK	8	10	Ramp (1.8-2.1 MPa)	Single point sonications
8	SBPK	10	10	Ramp (1.8-2.1 MPa)	Single point sonications

**Table 2:** Description of histology grades

Grade	Description
0	No damage on all histology levels
1	Very minor damage - 1 to a few very small clusters of red blood cells on at least 1 histology level
2	Moderate (more significant) damage - 1 larger cluster of red blood cells or a greater number of small clusters of red blood cells on at least 1 histology level
3	Extensive damage - A large number of clusters of red blood cell extravasation, with or without pooling on at least 1 histology level.

**Table 3:** Summary of results for BSCBO, tissue damage and acoustic emissions

Pig ID	Pulse Type	Location	Evans Blue Extravasation	MRI Enhancement	Macroscopic Damage	Histology Score	Subharmonic
3	Sine		Y		Y	3	Y
4	Sine		Y		Y	3	Y
	SBPK		Y		N	2	Y
5	SBPK	1	Y		N	1	Y
	SBPK	2	Y		N	1	Y
6	SBPK	1	Y		N		Y
	SBPK	2	Y		N		Y
	SBPK	3	N		N		N
	SBPK	4	Y		N		Y
7	SBPK	1	Y		N		
	SBPK	2	N		N		N
	SBPK	3	N		N		Y
	SBPK	4	Y		N		Y
	SBPK	5	Y		N		Y
	SBPK	6	N		N		Y
	SBPK	7	N		N		N
	SBPK	8	Y		N		Y
8	SBPK	1	N	N	N	2	Y
	SBPK	2	Y	Y	N	1	Y
	SBPK	3	Y	Y	N	1	Y
	SBPK	4	N	N	N	1	Y
	SBPK	5	Y	Y	Y	3	Y
	SBPK	6	N	Y	N	3	Y
	SBPK	7	Y	Y	Y	3	Y
	SBPK	8	Y	Y	N	2	Y
	SBPK	9	Y	Y	N	2	Y
	SBPK	10	Y	Y	Y	3	Y

**Table 4:** CT derived vertebral dimensions

		Vertebral dimensions [mm]	
		Spinal canal depth	Spinal canal width
		*µ*±*σ*	*µ*±*σ*
Porcine	T9	10.5±0.6	14.4±2.2
	T10	9.4±1.2	14.1±2.1
	T11	9.6±0.6	15.2±2.4
	T12		16.3±3.1
	T13		15.8±3.4
	T14		15.7±2.9
	T15		16.4±0.7
Human	T7	14.9±0.8	16.2±0.9
	T8	15.5±1.5	16.4±0.4
	T9	16.9±1.5	16.4±1.1
	T10	17.9±1.9	17.0±0.8
	T11	23.6±0.7	19.5±0.3
	T12	20.2±1.9	18.4±0.5

**Table 5:** CT derived densities of porcine and human vertebrae using the top 5% of CT voxels for each vertebra

		CT derived density [kgm^-3^]			
		Vertebral Body		Posterior elements	
		*µ*±*σ*	Min-Max	*µ*±*σ*	Min-Max
Porcine	T9	1582±89	1488-2043	1795±84	1695-2208
	T10	1494±68	1428-1847	1639±102	1528-2168
	T11	1570±80	1488-2046	1665±86	1565-2012
	T12-T15			1616±80	1537-2203
Human	T7	1705±167	1553-2604	2352±105	2217-2790
	T8	1684±123	1560-2387	2398±105	2260-2869
	T9	1697±113	1569-2375	2391±114	2240-2872
	T10	1779±145	1616-2482	2431±114	2280-2907
	T11	1736±146	1582-2468	2442±100	2304-2902
	T12	1741±146	1587-2445	2409±102	2270-2830

## Abbreviations

BBB: Blood-brain barrier
BBBO: Blood-brain barrier opening
BSCB: Blood-spinal cord barrier
BSCBO: Blood-spinal cord barrier opening
CNS: Central nervous system
CT: Computed tomography
EBD: Evans blue dye
FLAIR: Fluid-attenuated inversion recovery
FUS: Focused ultrasound
GRE: Gradient echo
H&E: Hematoxylin and eosin
IM: Intramuscular
IV: Intravascular
L1: 1^st^ lumbar vertebra
MB: Microbubble
MRI: Magnetic resonance imaging
PI: Pulse inversion
PRF: Pulse repetition frequency
PVDF: Polyvinylidene difluoride
PZT: Lead zirconium titanate
SBPK: Short burst, phase keying
SNR: Signal-to-noise ratio
T12: 12^th^ thoracic vertebra
